# Link between serum lipid signature and prognostic factors in COVID-19 patients

**DOI:** 10.1038/s41598-021-00755-z

**Published:** 2021-11-04

**Authors:** Michele Dei Cas, Sara Ottolenghi, Camillo Morano, Rocco Rinaldo, Gabriella Roda, Davide Chiumello, Stefano Centanni, Michele Samaja, Rita Paroni

**Affiliations:** 1 Department of Health Sciences, Università degli Studi di Milano, via A. di Rudinì 8, Milan, Italy; 2 Department of Pharmaceutical Sciences, Università degli Studi di Milano, Milan, Italy; 3 Respiratory Unit, San Paolo University Hospital, ASST Santi Paolo e Carlo, Milan, Italy; 4 Department of Anesthesia and Intensive Care, San Paolo University Hospital, ASST Santi Paolo e Carlo, Milan, Italy

**Keywords:** Prognostic markers, Lipidomics

## Abstract

Although the serum lipidome is markedly affected by COVID-19, two unresolved issues remain: how the severity of the disease affects the level and the composition of serum lipids and whether serum lipidome analysis may identify specific lipids impairment linked to the patients' outcome. Sera from 49 COVID-19 patients were analyzed by untargeted lipidomics. Patients were clustered according to: inflammation (C-reactive protein), hypoxia (Horowitz Index), coagulation state (D-dimer), kidney function (creatinine) and age. COVID-19 patients exhibited remarkable and distinctive dyslipidemia for each prognostic factor associated with reduced defense against oxidative stress. When patients were clustered by outcome (7 days), a peculiar lipidome signature was detected with an overall increase of 29 lipid species, including—among others—four ceramide and three sulfatide species, univocally related to this analysis. Considering the lipids that were affected by all the prognostic factors, we found one sphingomyelin related to inflammation and viral infection of the respiratory tract and two sphingomyelins, that are independently related to patients' age, and they appear as candidate biomarkers to monitor disease progression and severity. Although preliminary and needing validation, this report pioneers the translation of lipidome signatures to link the effects of five critical clinical prognostic factors with the patients' outcomes.

## Introduction

Severe Acute Respiratory Syndrome Coronavirus 2 *(*SARS-CoV-2), a positive-sense single-stranded RNA virus, is responsible for COVID-19 disease, which mainly affects the respiratory tract^1^. COVID-19 results in a plethora of symptoms that contribute independently to the severity of the infection. Although most patients infected by SARS-CoV-2 experience very mild to moderate illness, in some cases the infection leads to severe symptoms that eventually require hospitalization with a high mortality rate. The symptoms include respiratory failure, with a marked decrease in the oxygen partial pressure/inspired oxygen fraction ratio (Horowitz index or P/F), and shock accompanied by multi-organ dysfunction^2^.

Laboratory findings in the sera obtained from severely ill COVID-19 patients often display high levels of biomarkers, e.g. C-reactive protein (CRP), D-dimer (DD) and excessive cytokines release (also known as cytokine storm), linked to systemic hyper-inflammation, some of which are commonly found in patients with acute respiratory distress syndrome (ARDS)^3^. However, despite some similarities, ARDS and severe COVID-19 represent separate clinical entities in terms of lung compliance and endothelial inflammation^4^. Nevertheless, several observations converge in believing that COVID-19 targets multiple organs (lungs, liver, kidney, brain, testis, and intestine), impairing their function and leading to multi-organ injury^3^. Like many other viruses, SARS-CoV-2 triggers several pathways linked to both the metabolome and the lipidome. Consequently, it is expected that small-sized metabolites become essential to support virus replication by providing building blocks to assemble the viral nucleic acids, proteins, and membrane^5,6^. As for the lipidome signature, it has been shown that SARS-CoV-2 infection mobilizes the host free fatty acids pool to support viral capsid-associated membrane formation^7^. Many viruses can profoundly alter the host lipidome and exploit the host energy resources to support their replication, thereby causing marked changes in the plasma/serum lipidome^8,9^.

To fill the knowledge gap regarding the composition of the serum lipids set in COVID-19 patients, the main goal of the present study is to investigate the changes occurring within the disease severity. The aim is two-fold: (1) to unravel how the lipidome is affected by progressive impairment of the most critical prognostic factors employed to assess the severity of COVID-19 patients; and (2) to test whether the patients' outcome is marked by specific lipidome signatures independently of these prognostic factors.

At first, by employing an untargeted approach based on an up-to-date liquid chromatography tandem high-resolution mass spectrometry (LC-HR-MS) lipid screening technology, we identify the most frequent lipidome alterations as a function of the severity of the prognostic factors. Then, we used the same opportunity to assess which lipidome signature is primarily occurring in COVID-19 patients as a function of 7-day mortality. We reasoned that comparing the two lipidome signatures may give a unique insight into the identification of those lipids species that mark not only the severity, but also the patients' outcome, thereby identifying those who most require aggressive therapy.

## Results

### Patients

Table 1 reports the patients' main data at the time of blood sampling obtained within 48 h after hospitalization. Of these, twelve patients faced fatality within 7 days after hospitalization. Here, we selected the following markers: (1) serum CRP, (2) the Horowitz Index (P/F), (3) serum creatinine (CR), and (4) serum DD, hallmarks of the prognostic factors inflammation, hypoxia, kidney function and coagulation state, respectively. The fifth prognostic factor was age.

**Table 1:** Main data of recruited patients (mean ± SD).

	Reference values	All	Survivors	Deceased	p value
N (male)		50 (43)	38 (33)	12 (10)	
***Age (years)***		*60* ± *13*	*57* ± *12*	*70* ± *11*	*0.003*
pH	7.37–7.43	7.44 ± 0.05	7.44 ± 0.03	7.41 ± 0.07	0.059
**paCO**_**2**_** (mmHg)**	36–46	41.1 ± 7.0	39.6 ± 5.5	45.9 ± 9.3	0.008
paO_2_ (mmHg)	73–99	114 ± 73	125 ± 81	77 ± 12	0.053
spO_2_ (%)	95–98	97 ± 2	98 ± 2	96 ± 2	0.072
FiO_2_	0.19–0.21	0.56 ± 0.20	0.54 ± 0.21	0.65 ± 0.10	0.090
**Horowitz Index**	> 400	209 ± 105	238 ± 105	118 ± 21	0.001
BE (mEq/L)	− 2, + 2	3.8 ± 3.6	3.6 ± 3.4	4.4 ± 4.4	0.509
Hb (g/dL)	12–15.2	12.7 ± 1.6	12.7 ± 1.6	12.5 ± 1.5	0.75
WBC (× 10^3^/μL)	3.5–10	9.7 ± 7.0	8.9 ± 7.4	12.5 ± 4.0	0.15
**Creatinine (mg/dL)**	0.52–1.04	0.91 ± 0.52	0.82 ± 0.53	1.16 ± 0.39	0.050
**Bilirubin (mg/dL)**	0.2–1.3	0.9 ± 0.7	0.7 ± 0.3	1.6 ± 1.2	0.0003
**CRP (mg/L)**	< 10	73 ± 44	62 ± 41	114 ± 27	0.0006
**LDH (U/L)**	120–246	399 ± 162	321 ± 23	542 ± 162	0.0001
**D-dimer (ng/mL)**	< 270	4213 ± 13,858	999 ± 3059	14,175 ± 25,627	0.007

The parameters that differentiate for the patients' outcomes are in bold. The prognostic factors selected to cluster patients in this study are in italics. p values were determined by Student t-test for unpaired data.

### Lipid alterations

Untargeted lipidomics analysis on serum of COVID-19 patients identified about 1500 lipid species. The striking serum lipid profile differences that emerged when comparing COVID-19 patients with healthy age-matched controls (n = 10), suggest that such differences are related to the disease state rather than the patients' age or the preceding lifestyle (Supplementary Figure S1).

### Lipidome vs. inflammation

**Figure 1:**
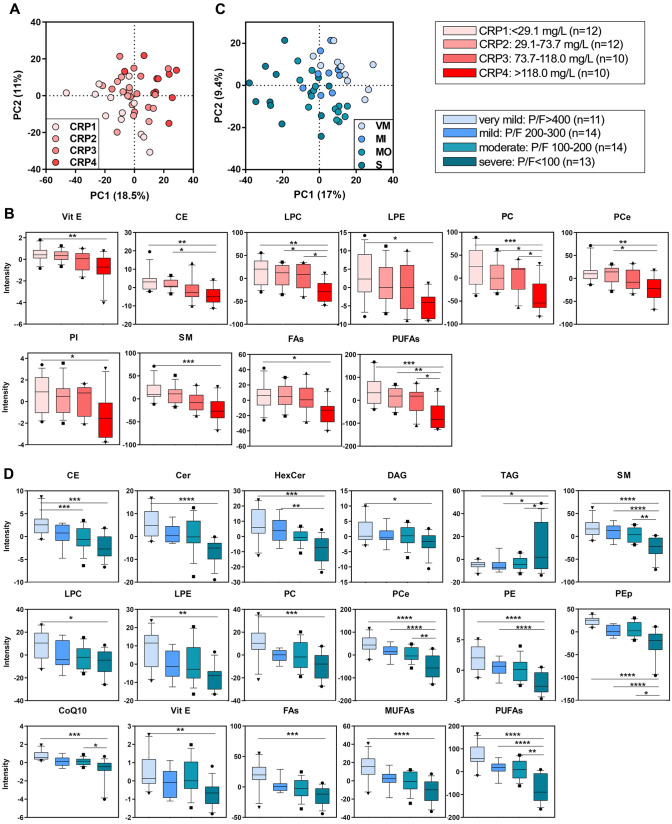
Discriminant analysis (score plot) of the lipidome as a function of inflammation (**A**) and hypoxia (**C**). Lipid classes with a statistical significance are shown in boxplots in function of increasing inflammation (**B**) and hypoxia (**D**). Boxes: 25th–75th percentiles; lines: 10th–90th percentiles; crossing lines: median values; separate points: outliers. Statistical tests were performed by one-way ANOVA and the Bonferroni post hoc test.

For this analysis, COVID-19 patients were grouped in four quartiles according to CRP: CRP1 (< 29.1 mg/L, n = 12); CRP2 (29.1–73.7 mg/L, n = 12); CRP3 (73.7–118.0 mg/L, n = 10), and CRP4 (> 118.0 mg/L, n = 10). The discriminant analysis (PLS-DA), used to discriminate the lipidomic profiles among the four groups, showed a separation of 18.5% on principal component (PC1). The PC1 represents the new dimension in which the initial variables are compressed and represents the maximum of the separation that can be reached within these clusters and variables (Fig. 1A). The VIP scores derived from PLS-DA were used for ranking the discriminating features, taking a cut-off value > 1.5. From this cluster, it emerged that 240 lipid species, hereinafter called discriminant lipids, marked univocally the differences among the four groups as far as inflammation was concerned. Univariate analysis was performed to validate this dataset and to test whether the trend in the discriminant lipids was consistent with the CRP level and with the severity of the inflammation. Lipid classes that have a statistically significant modulation (Fig. 1B) include phospholipids, sphingomyelins (SM), CE, the antioxidant vitamin E (Vit. E) and the anti-inflammatory bulk of lipids containing polyunsaturated fatty acids (PUFAs).

### Lipidome vs. hypoxia

For this analysis, COVID-19 patients were grouped in four quartiles according to P/F as a marker of the respiratory failure severity: very mild (VM, P/F > 400, n = 11), mild (MI, P/F 200–300, n = 9), moderate (MO, P/F 100–200, n = 14), and severe (S, P/F < 100, n = 13). Critically intubated patients were included in the S group independently of P/F (P/F = 140 ± 41). PLS-DA resolved mild from severe cases with a separation of 17% on PC1 (Fig. 1C), with 264 discriminant lipids species that marked the differences among the four groups as far as hypoxia was concerned. Univariate analysis showed depletion of the antioxidants Vit. E, Coenzyme Q10 (CoQ10) and sterols, sphingolipids and phospholipids, especially the plasmalogens vinyl-linked phosphatidylethanolamines (PEp), with decreasing P/F (Fig. 1D). By contrast, triacylglycerols (TAG) were increased with the severity of hypoxia.

### Lipidome vs. coagulation state

**Figure 2:**
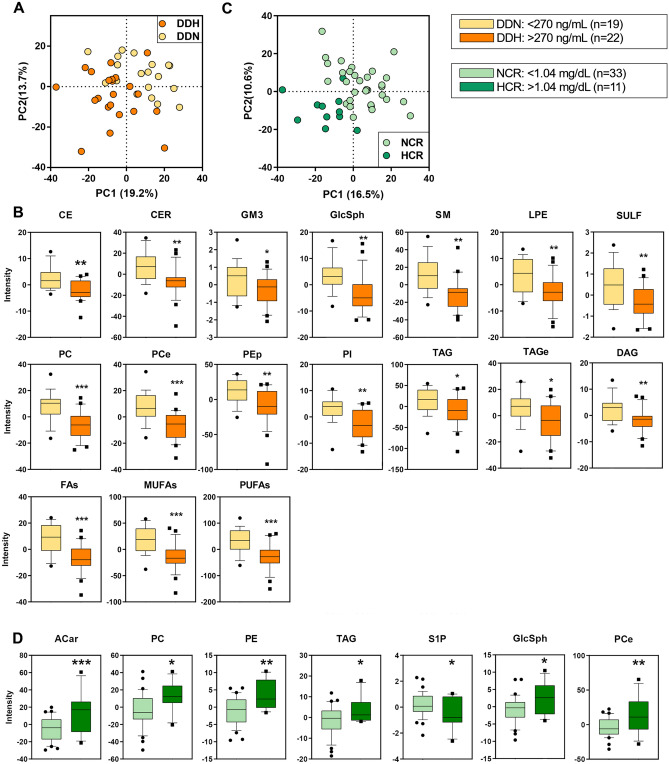
Discriminant analysis (score plot) of the lipidome as a function of coagulation (**A**) and kidney function (**C**). Only lipid classes with a statistical significance are shown in boxplots in function of increasing in D-dimer (**B**) and creatinine (**D**) concentrations. Boxes: 25th–75th percentiles; lines: 10th–90th percentiles; crossing lines: median values; separate points: outliers. Statistical tests were performed by univariate t-test.

COVID-19 patients were clustered in two groups according to the level of DD, a marker of thrombotic disease and microvascular coagulopathy: normal (DDN, < 270 µg/L, n = 19) and high (DDH, > 270 µg/L, n = 22). PLS-DA discriminated DDN and DDH patients by 19.2% on PC1 (Fig. 2A). From this analysis, it emerged that 248 discriminant lipids marked univocally the differences among the two groups on the basis of the coagulation state. Figure 2B shows that all the discriminant lipids were depleted to a larger extent in DDH than in DDN.

### Lipidome vs. kidney function

For this analysis, COVID-19 patients were clustered into two groups according to serum CR, a marker of the glomerular filtration rate and kidney function: normal (NCR, < 1.04 mg/dL, n = 33) and high (HCR, > 1.04 mg/dL, n = 11). PLS-DA discriminated NCR and HCR patients by 16.5% on PC1 (Fig. 2C). It emerged that 219 discriminant lipids marked univocally the differences among the two groups as far as kidney function was concerned. Among the discriminant lipids, several classes, i.e., phosphatidylcholines (PC), phosphatidylethanolamines (PE), acylcarnitines (ACar), free cholesterol (Chol) and glycosphingolipids (GlcSph), were increased in HCR with respect to NCR (Fig. 2D). Interestingly, the pro-survival sphingosine-1-phosphate (S1P) decreased in HCR and never showed a statistical significance in any previous cluster except for kidney function.

### Lipidome vs. age

**Figure 3:**
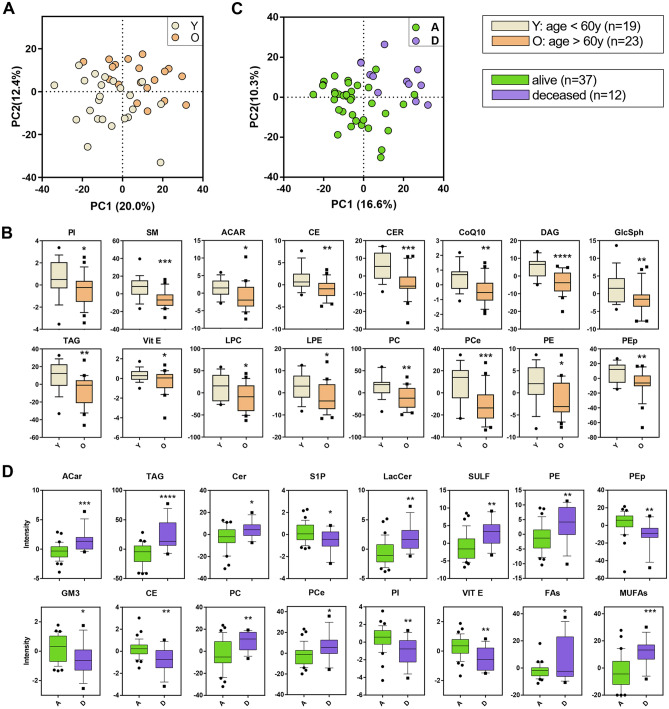
Discriminant analysis (score plot) of the lipidome as a function of age (**A**) and outcome (**C**). Only lipid classes with a statistical significance in the groups of each clusterization are shown in boxplots: age in (**B**) and outcome in (**D**). Boxes: 25th–75th percentiles; lines: 10th–90th percentiles; crossing lines: median values; separate points: outliers. Statistical significance: univariate t-test.

Patients were clustered in two groups according to age at admission: relatively younger (Y, < 60  year-old, n = 19) and older (O, > 60 year-old, n = 23). PLS-DA discriminated Y and O patients by 20% on PC1 and 12.4% on PC2 (Fig. 3A). Specifically, 241 lipids univocally marked the differences among the two groups. Univariate analysis shown in Fig. 3B reveals a particular depletion of all the major lipid classes in O.

### Lipidome vs. outcome

The same approach above reported for prognostic factors was performed to cluster patients into survivors (A, n = 37) and deceased within 7 day after hospitalization (D, n = 12). Both groups displayed two distinct lipidomic patterns (PC1, 16.6%, Fig. 3C) that only partially overlapped with those related to prognostic factors clustering. The lipid signature in D highlighted the presence of 210 discriminant lipids, particularly (Fig. 3D): ACar, Ceramides (Cer), GlcSph, sulfatides (SULF), PE, PC, ether-linked phosphatidylcholines (PCe), and TAG rose, while CE, S1P, GM3, PEp, phosphatidylinositols (PI) and Vit. E were decreased in D with respect to A. S1P displayed the same behavior observed in renal function.

We finally identified 29 lipid species exclusively linked to fatal outcome by subtracting from the set of 210 VIPs lipids, all the common species previously established as a discriminant in the other five prognostic factors (Fig. 5C). The lipid species that accumulated in the sera of D patients include Cer, SULF, SM, PC, monoacylglycerols (MAG) and TAG, while those that diminished include GM3 24:0, the MAG 20:1 and PCe 40:8.

### Prognostic factors: common findings

**Figure 4:**
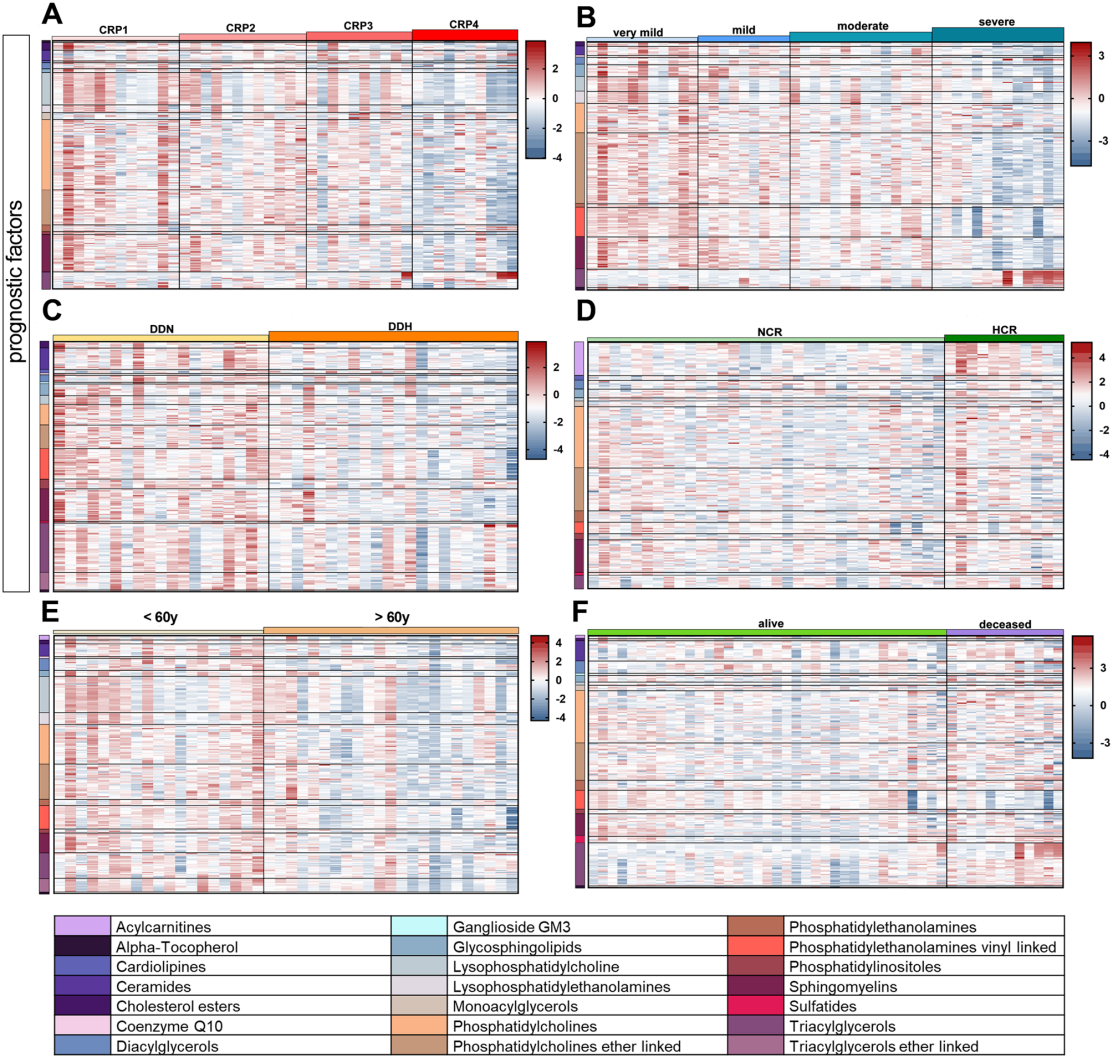
Heatmaps of the lipids highly correlated with each prognostic factor—(**A**) inflammation, (**B**) hypoxia, (**C**) coagulation state, (**D**) kidney function and (**E**) age—and with the outcome (**F**) chosen within those with a Variance Importance in Projection (VIP) score superior than 1.5, ordered by lipid classes.

In Fig. 4, discriminant lipids are visualized by heatmaps and ordered according to their subclasses. The panels report the discriminant lipids according to the prognostic factors inflammation (Fig. 4A), hypoxia (Fig. 4B), coagulation state (Fig. 4C), kidney function (Fig. 4D), age (Fig. 4E) and the outcome (Fig. 4F). Serum lipids were progressively depleted with rising inflammation, hypoxia and age.

**Figure 5:**
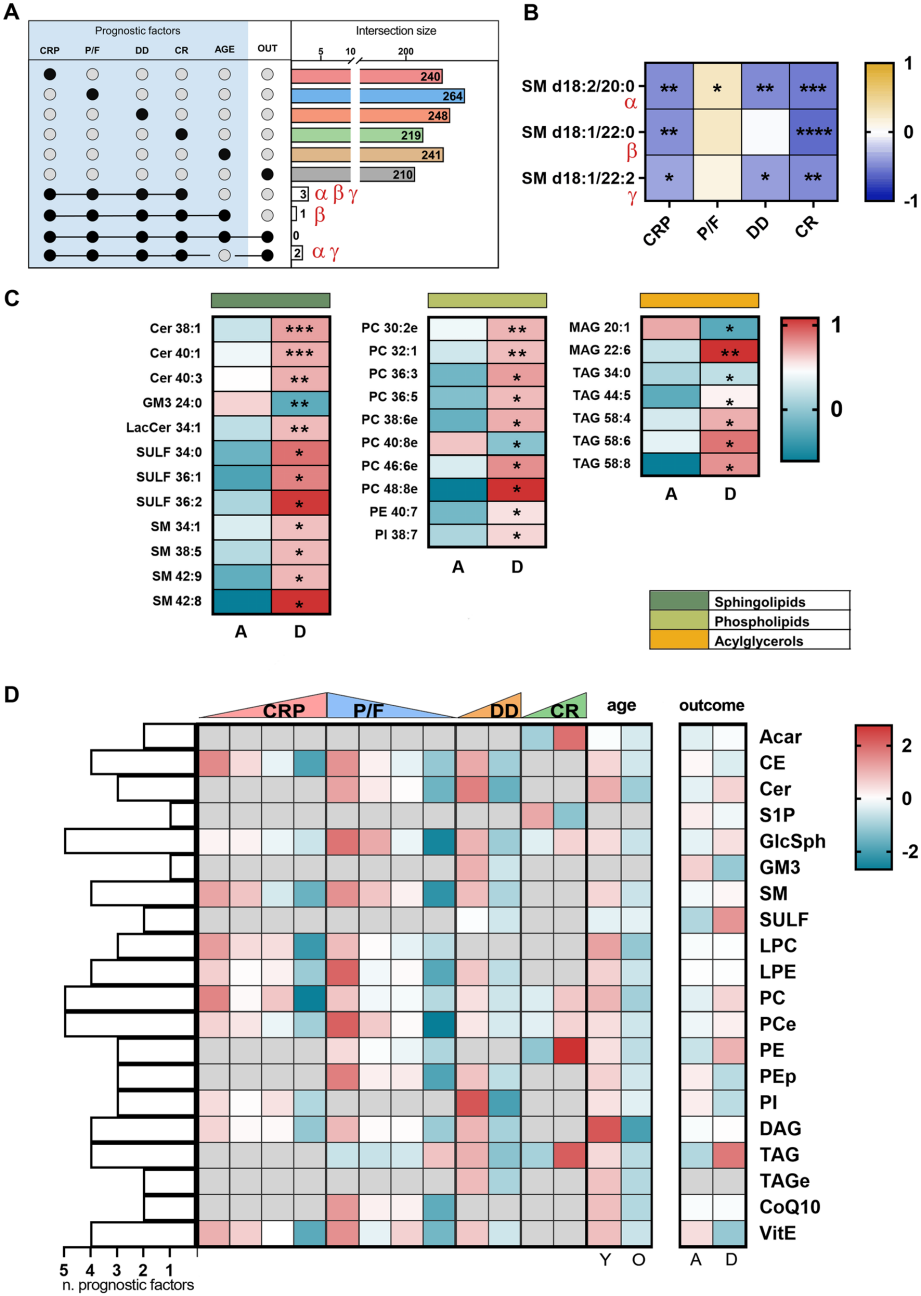
In (**A**) and (**B**), intersection size graph and Pearson correlation of lipids commons in the selected prognostic factors and outcome. (**C**) Heatmap of lipids associated uniquely with death. In (**D**), the heatmaps on the left show the lipid classes perturbed by the severity of the disease according to five prognostic factors. Those on the right report the alteration of the lipid according to the outcome. A grey square denotes no significant alteration of that class in that prognostic factor. On the left side, the bars indicate the number of the prognostic factors each lipid class is involved in.

Finally, looking for specific lipid signatures common to the investigated prognostic factors, we found three sphingomyelins (SM d18:2/20:0, SM d18:1/22:0 and SM d18:1/22:2) common to CRP, P/F, DD, CR (Fig. 5A, Supplementary Figure S2). These species are negatively related to CRP and CR, while SM d18:2/20:0 is related to all the prognostic factors (Fig. 5B).

By adding age to this analysis, only SM d18:1/22:0 remained a common lipid. Moving to intersect the lipids set in prognostic factors with outcome, no common species were found (Fig. 5A). Removing the age set from this analysis, two SMs, (d18:2/20:0 and SM d18:1/22:2) remarkably emerged as a specific link between prognostic factors and outcome. Neither SM d18:2/20:0 nor SM d18:1/22:2 is affected by the patients' age (Fig. 5A).

## Discussion

This study highlights the lipidomic signature as a function of both a panel of prognostic factors related to the disease severity and the outcome in consecutive COVID-19 patients enrolled in the period May–November 2020 in the Intensive Care Unit and the Pneumology Department of ASST Santi Paolo e Carlo (Milan, Italy). As we are facing a disease with unprecedented complexity and unclear pathogenesis, we believe that the scientific community may benefit from this preliminary study where we correlate the lipidome signature underlying the effect of prognostic factors with the outcome.

Our first purpose was to identify the most suitable prognostic factors. By ranking the disease progression through the engagement of a few clinical variables predicted to influence the outcome, the identified markers were assumed to recapitulate the development of inflammation, hypoxia, coagulation, and kidney function, as well as the independent effects of the patients' age. Although other potentially discriminating prognostic factors (blood pH, leukocytes, LDH, and base excess) could also be engaged, the selected markers were found to converge toward a reliable lipidome signature.

Although the serum lipidome is perturbed differently by each prognostic factor (Fig. 4), a few common features are nevertheless distinguished. Assuming the 20 major classes of lipids outlined in Fig. 5D, serum lipids tended to diminish with the advancement of inflammation, hypoxia, coagulation state and age (CE, SM, lysophosphatidylethanolamine (LPE), diacylglycerols (DAG), Vit. E, GlcSph, PC, PCe), and to increase with declining kidney function (GlcSph, PC, PCe, ACar, PE). Previous studies showed marked dyslipidemia in COVID-19 patients^6,10–13^. This drop in serum lipids may be associated with the multi-organ deterioration caused by worsening inflammation, oxygenation impairment and hyper-coagulation^12,14^. The genesis of this phenomenon may derive from the evolutionary advantage for coronaviruses to use components of the host cells machinery, specifically the endoplasmic reticulum and the Golgi compartments, during their replication process^11,15^. Indeed, SARS-CoV-2 uses host-derived lipid membranes when building the capsid, which helps the virus to hide from the host immune system especially during the first phases of infection, thereby worsening the disease with pivotal outcomes as lymphopenia, cytokine storm and pneumonia, typical symptoms of severe COVID-19^11,16^. However, the goal of this study was not to verify this trend, but rather to investigate in detail the lipidomic signature underlying dyslipidemia.

The data here reported show that worsening COVID-19 infection profoundly alters the serum lipidome consistently with a metabolic shift towards lipolysis^17^. The Cer catabolite S1P is well-known to be linked to hypoxia as well as to immunological and inflammatory reactions^18,19^. Its depletion was associated with COVID-19 cytokine storm, endothelial barrier dysfunction and altered immune response^6,20^. S1P's protective effect on hypoxia-induced oxidative stress and endothelial dysfunction results from its proliferative, anti-apoptotic effects^21^, which forms the rationale for its use as a therapy for hypoxia-related damage^22^. Moreover, the often-observed depletion of Vit. E, a component of the antioxidant defense, previously linked to alterations in immune responses and viral pathogenicity^23^, may be associated with an uncontrolled immune response in COVID-19 patients, thereby suggesting its beneficial effects in strengthening the host resistance against respiratory infections^23^. Coherently with these observations, an ongoing trial is currently testing the role of Vit. E along with other vitamins to reduce severity and mortality rate of COVID-19 patients under intensive care^24^. In addition, mitochondrial CoQ10 is reduced with progressive oxidative stress, hypoxia, or decreased P/F^25^. The same behavior is appreciated in ether lipids (i.e. PCe, PEp) as they may protect the phospholipid cell membrane against oxidation stress acting as reductants^26^.

Then, our goal was to assess how the selected prognostic factors affected the lipidome signature. This analysis produced a panel of lipid species that were impaired in the same direction by the prognostic factors. When the lipidome signatures associated with CRP, P/F, DD and CR (Fig. 5A) were intersected, three lipid species emerged as shared among all the tested clusterization: the structures of two of them correspond to that of SMs with two saturated fatty acid chains of 20 or 22 carbon atoms, while the third corresponds to SM with one polyunsaturated chain of 22 carbon atoms (22:2). Remarkably, d18:1/20:0 was recently linked to the insurgence of Type 2 diabetes^27^. SM depletion may be linked to enhanced activity of serum acid sphingomyelinase, which has been reported to increase in systemic inflammatory diseases and respiratory tract infection^28^.

Afterward, we focused on the outcome (7-day mortality) to test whether the lipidome signature characteristic of fatality mirrored the same lipids species that were identified in the first and second steps. We reasoned that assessing the occurrence of lipid species in either selection would considerably impact the identification of novel reliable lipid biomarkers to predict the disease progression. The analysis shown in Fig. 5C identified 29 lipid species whose changes are exclusively shared in the patients who faced fatal outcomes. The critically ill D patients display a peculiar lipidome signature characterized by increased levels of four Cer and three SULF species. The rise in pro-apoptotic Cer is justified by similar changes observed in fragile patients at high coronary risk^29^, as well as in hypoxia- and inflammation-linked pathological conditions such as chronic obstructive pulmonary disease^30^. Such a rise may reflect either the increased activity of sphingomyelinase observed in ARDS patients with worse prognosis^31^ or increased de-novo sphingolipid biosynthesis^32^. By contrast, the acid sphingolipid GM3 24:0 showed a significant decrease. The reduction of GM3 24:0 in plasma may be counterbalanced by the segregation of GM3 into the exosomes, as proposed in a study that highlighted the involvement of CD4+ T cells in COVID-19^11^. In addition to this speculation, it has been shown that exosomes enriched in GM3 may derive from immunocompetent cells^33^. Nonetheless, circulating exosomes may contain other bioactive lipids, whose function could be associated with signaling from a secreting to a receiving tissue. Furthermore, the alteration of exosome-mediated lipid trafficking could be part of major pathological conditions^34,35^. We also observed a puzzling TAG elevation in D patients, apparently in contrast with the state of increased lipolysis with the infection severity. Although the cause is unclear, it may be matched to liver pathologic alterations that lead to hepatic steatosis, which in turn stimulates the conversion of white to brown adipose tissue with enhanced lipogenesis^17^.

In addition to a panel of 29 lipids uniquely linked to the outcome, we recognized two SMs that are simultaneously affected also by the selected prognostic factors: d18:2/20:0 and d18:1/22:2. Noteworthily, these SMs are unrelated to the patients' age (Fig. 5A,B), therefore they are predicted to mark disease severity and outcome, thereby representing a specific signature of COVID-19 disease.

The present study has some limitations. (1) Analyses were performed in a small set of COVID-19 patients, in which the D sub-group was disproportionally represented (12/49). (2) The group with severe respiratory disease included both intubated and non-intubated patients, which may have confounded some of the findings. (3) We have intentionally restricted the wide range of prognostic factors^36^ to five, to maintain our focus. (4) We did not include in this study a comparison with non-COVID-19 patients admitted to Intensive and Sub-Intensive Care Units for other respiration-related illnesses.

Several reports have been published in the last year covering the lipid signature(s) left by COVID-19 and the lipid spectrum shifts related to disease severity^6,10–12,37–39^. Despite inherent difficulties to match data obtained in different populations and with different analytical instruments, among other variables, it appears that they tend to converge in a kaleidoscopic pattern of lipids alterations that include upregulation of TAG, ACar, Cer, and downregulation of glycosphingolipids^40^. Thus, common accordance is yet far from being reached.

The data described here likely constitute the first report on the comprehensive serum lipidome signature in COVID-19 patients as a function of selected markers (serum CRP, the P/F ratio, serum DD, serum CR and age) that recapitulate clinical features related to inflammation, oxygenation, coagulation and renal function. The correlation between the lipid classes affected by the selected prognostic factors with those that determine the fatal outcome and depend on the patients' age yields a panel of lipids that may address the perspective of using lipidomic-linked technology as a prognosis tool in the current COVID-19 pandemic to identify novel potential biomarkers. It appears that three SMs are affected by the selected prognostic factors. As two of these SMs are also shared in the panel of lipid species clustered around the outcome, they may be regarded as potential biomarkers to monitor the disease progression. At present, we are unable to state the real diagnostic advantages of evaluating the lipid signature concerning routine clinical assays. However, the differential lipids assessment may nevertheless be taken as an adjuvant tool to monitor disease progression and severity.

## Material and methods

### COVID-19 patients

COVID-19 positive consecutive patients (n = 50) with pneumonia were recruited, between May and November 2020, from the Intensive Care Unit and the Pneumology Department of ASST Santi Paolo e Carlo (Milan, Italy), within 48 h from the hospitalization. Pneumonia in the recruited patients was defined as a respiratory failure (spO_2_ < 90% or PO_2_ < 60 mmHg at ambient air or specific radiological findings). All patients had a molecular diagnosis of SARS-CoV-2 infection^41^ with evidence of pneumonia based on chest X-rays or computed tomography. All the patients received corticosteroids and low-molecular-weight heparin. Exclusion criteria included age < 18 years, recent transfusions and chronic kidney failure. One patient was excluded for severe renal insufficiency, which caused aberrancy in lipidome. Therefore, the final number of COVID-19 patients enrolled is 49. Control samples for lipidomics (n = 10) were matched on age (65 ± 4 years) (Table 1) and sex with enrolled patients. Whole blood was collected, and sera were obtained as soon as possible by using a refrigerated centrifuge at 4 °C. Aliquots were prepared keeping the samples in ice, and stored at − 20 °C until analysis.

### Ethics approval and consent to participate

All experimental protocols regarding human materials were conducted according to the Declaration of Helsinki and were approved by The Milan Area 1 Ethics Committee, who has approved the execution of the blood sampling for this study on ARDS patients and healthy subjects (prot. 5098/2020, 2019/ST/144). The same committee later approved the extension of the protocol to COVID-19 patients. Informed consent for study participation was gathered from all subjects before enrollment or from the subject's legally authorized representative if the subjects could not provide it.

### Chemicals and reagents

The chemicals acetonitrile, 2-propanol, methanol, chloroform, formic acid, ammonium acetate and butylated hydroxytoluene were purchased from Sigma-Aldrich (St. Louis, MO, USA). All aqueous solutions were prepared using purified water at a Milli-Q grade (Burlington, MA, USA).

### Untargeted lipidomics

Sera (25 µl) were extracted by methanol/chloroform mixture^42^. The addition of butylated hydroxytoluene (BHT) during sample preparation avoided unspecific oxidation. LC–MS/MS consisted of a Shimadzu UPLC coupled with a Triple TOF 6600 Sciex (Concord, ON, CA) equipped with Turbo Spray IonDrive. All samples were analyzed in duplicate in positive mode with electrospray ionization. Spectra were contemporarily acquired by full-mass scan from *m/z* 200–1500 and top-20 data-dependent acquisition from *m/z* 50–1500. Declustering potential was fixed to 50 eV, and the collision energy was 35 ± 15 eV. The chromatographic separation was reached on a reverse-phase Acquity CSH C18 column 1.7 μm, 2.1 × 100 mm (Waters, Franklin, MA, USA) equipped with a precolumn by a gradient between (A) water/acetonitrile (60:40) and (B) 2-propanol/acetonitrile (90:10), both containing 10-mM ammonium acetate and 0.1% of formic acid^42^.

### LC-HR-MS data processing

The spectra deconvolution, peak alignment and sample normalization were attained using MS-DIAL (ver. 4.0). MS and MS/MS tolerance for peak profile was set to 0.01 and 0.05 Da, respectively. Identification was achieved matching spectra with LipidBlast database or in-house built mass spectral library. Intensities of analytes were normalized by Lowless algorithm and those with a CV% superior to 30% in the QC pool sample were excluded.

### Statistics and data visualization

Graphs and statistical analyses were prepared with GraphPad Prism 7.0 (GraphPad Software, Inc, La Jolla, California, USA), and with MetaboAnalyst 4.0. Univariate statistical analysis was performed using one-way ANOVA with Bonferroni post hoc test for comparing lipid concentrations across different groups, whereas unpaired t-test was used for two-group comparison. For multivariate analysis, data were checked for integrity, filtered by interquartile range, log-transformed and auto-scaled. Partial least squares discriminant analysis (PLS-DA) was performed to increase the group separation and investigate the variables with a high Variance Importance in Projection score (VIP > 1.5). Pearson correlation analysis was performed to investigate the linear relationship between lipids and some continuous clinical variables (i.e. CRP, P/F, DD and CR) and associated p values were subsequently used. p values < 0.05 was considered statistically significant. Data are shown as mean ± SD.

## Supplementary Information

Supplementary Information.

## Data Availability

Metabolomics resources and data are available under the EMBL-EBI’s Terms of Use (https://www.ebi.ac.uk/about/terms-of-use) via the web https://www.ebi.ac.uk/metabolights/MTBLS3640.

## Abbreviations

ACar: Acylcarnitines
BE: Base excess
CE: Cholesterol esters
Cer: Ceramides
chol: Cholesterol
CoQ10: Coenzyme Q10
COVID-19: Coronavirus Disease
CR: Creatinine
CRP: C-reactive protein
DD: D-dimer
DHCer: Dihydroceramides
FAs: Saturated fatty acids
Gb3: Globotriaosylceramide
GlcSph: Glycosphingolipids
GM3: Gangliosides
HexCer: Hexosylceramides
LacCer: Lactosylceramides
LDH: Lactate dehydrogenase
LP: Lysophospholipids
LPC: Lysophosphatidylcholines
LPE: Lysophosphatidylethanolamines
MUFAs: Monounsaturated fatty acids
Horowitz Index, P/F: Oxygen partial pressure/inspired oxygen fraction
PC: Phosphatidylcholines
PCe: Ether-linked phosphatidylcholines
PE: Phosphatidylethanolamines
PEp: Vinyl linked phosphatidylethanolamines
PI: Phosphatidylinositoles
PLS-DA: Partial least squares discriminant analysis
PUFAs: Polyunsaturated fatty acids
S1P: Sphingosine-1-phosphate
SARS-CoV-2: Severe Acute Respiratory Syndrome-Coronavirus 2
SM: Sphingomyelins
SULF: Sulfatides
Vit E: Alpha-tocopherol
VIP: Variance Importance in Projection Score
