# Author Correction: Link between serum lipid signature and prognostic factors in COVID-19 patients

**DOI:** 10.1038/s41598-022-07501-z

**Published:** 2022-03-03

**Authors:** Michele Dei Cas, Sara Ottolenghi, Camillo Morano, Rocco Rinaldo, Gabriella Roda, Davide Chiumello, Stefano Centanni, Michele Samaja, Rita Paroni

**Affiliations:** 1 Department of Health Sciences, Università degli Studi di Milano, via A. di Rudinì 8, Milan, Italy; 2 Department of Pharmaceutical Sciences, Università degli Studi di Milano, Milan, Italy; 3 Respiratory Unit, San Paolo University Hospital, ASST Santi Paolo e Carlo, Milan, Italy; 4 Department of Anesthesia and Intensive Care, San Paolo University Hospital, ASST Santi Paolo e Carlo, Milan, Italy

Correction to: *Scientific Reports* 10.1038/s41598-021-00755-z, published online 04 November 2021

The original version of this Article contained an error in the Funding section.

“This research was funded by Dipartimento di Scienze della Salute, Università degli Studi di Milano (Piano di Sostegno alla Ricerca LINEA 2: Dotazione annuale per attività istituzionali with a project entitled Iron handling in patients exposed to acute and chronic hypoxia-FeOx) and Ministero dell'Istruzione, dell'Università e della Ricerca (Programma Nazionale di Ricerca in Antartide. Bando PNRA 25 maggio 2018, n. 1314 PNRA18_00071-F with a project entitled Concorde Impact of the Antarctic environments on human homeostasis, psychology, physiology and immunity”).

now reads:

“This research was funded by Dipartimento di Scienze della Salute, Università degli Studi di Milano (Piano di Sostegno alla Ricerca LINEA 2: Dotazione annuale per attività istituzionali within a project entitled “FeOx. Iron handling in patients exposed to acute and chronic hypoxia”, by Ministero dell'Istruzione, dell'Università e della Ricerca (Programma Nazionale di Ricerca in Antartide, PNRA18_00071-F within a project entitled “Concorde. Impact of the Antarctic environments on human homeostasis, psychology, physiology and immunity”), by Ministero dell'Istruzione, dell'Università e della Ricerca (FISR-COVID-19 Project FISR2020IP_01583, within a project entitled “HITCoA. Impact of Hypoxia, Iron Toxicity and oxidative stress on COvid19 Anemia”).”

The original Article has been corrected.

