# Supplementary material for: Link between serum lipid signature and prognostic factors in COVID-19 patients
Source: Sci Rep. 2021 Nov 4;11:21633. doi: 10.1038/s41598-021-00755-z (PMC8568966; doi:10.1038/s41598-021-00755-z)
Supplement: Supplementary file 1 — Supplementary Information. [file 41598_2021_755_MOESM1_ESM.pdf]

Supplementary materials.

Link between serum lipid signature and prognostic factors in COVID-19 patients

Running title: Serum lipidome in COVID-19 patients

Michele Dei Cas[1#], Sara Ottolenghi[1#], Camillo Morano[2], Rocco Rinaldo[1,3], Gabriella Roda[2], Davide Chiumello[1,4], Stefano Centanni[1,3], Michele Samaja[1*], Rita Paroni[1*]

[1] Department of Health Sciences, Università degli Studi di Milano, Milan, Italy

[2] Department of Pharmaceutical Sciences, Università degli Studi di Milano, Milan, Italy

[3] Respiratory Unit, San Paolo University Hospital, ASST Santi Paolo e Carlo, Milan, Italy

[4] Department of Anesthesia and Intensive Care, San Paolo University Hospital, ASST Santi Paolo e Carlo, Milan, Italy.

# the authors contribute equally to this study

*co-senior authors

*Corresponding authors: Rita Paroni, Associate Professor in Clinical biochemistry, rita.paroni@unimi.it, +390250323272, Department of Health Sciences, Università degli Studi di Milano, via A.di Rudinì 8, Milan, Italy

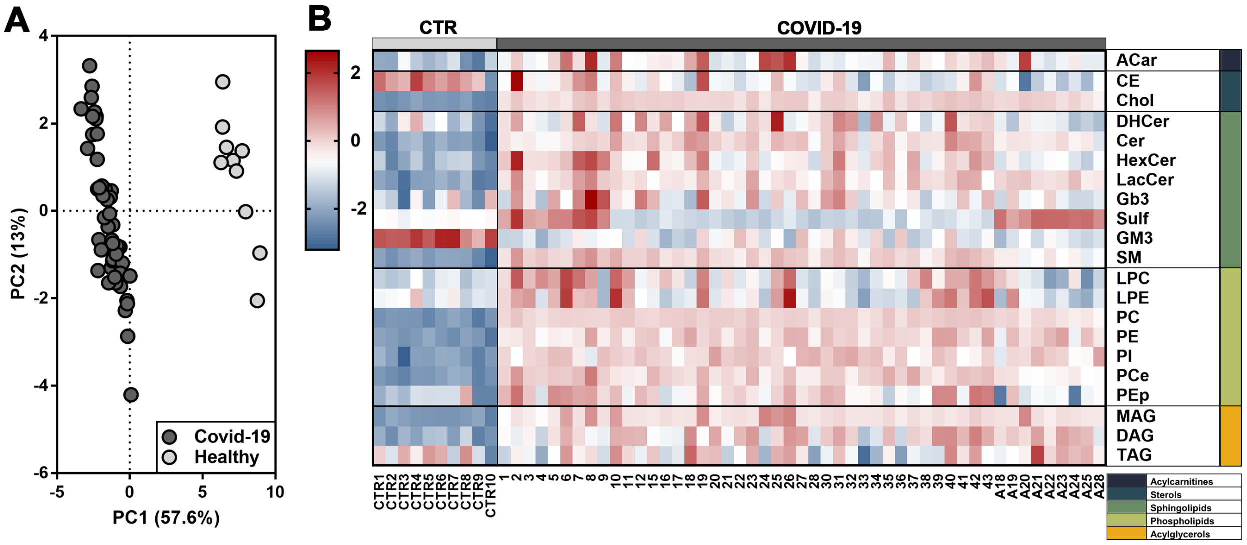

Figure S1. Serum lipid alteration related to symptomatic COVID-19 infection compared with healthy subjects. (**A**) Discriminant analysis (score plot) of the lipidome in function of the presence of symptomatic infection. The axes are ranked according to their importance in the group discrimination. In the x-axis, component 1 (PC1, 57.6%) represents the maximum of the separation that can be reached within these cluster and variables, whereas in the y-axis, component 2 (PC2, 13%) represents the direction that contains the most remaining variance. **(B)** Heatmap of the major serum lipids subclasses grouped per lipid categories coded by color on the left. The concentrations were autoscaled and log-transformed for visualization. The color-scale differentiates values as high (red), mean (white) and low (blue). Statistical significance was evaluated by unpaired t test. The concentrations of serum sulfatides, lysophospatidylethanolamines and triacylglicerols were not statistically different between healthy subjects and COVID-positive patients.

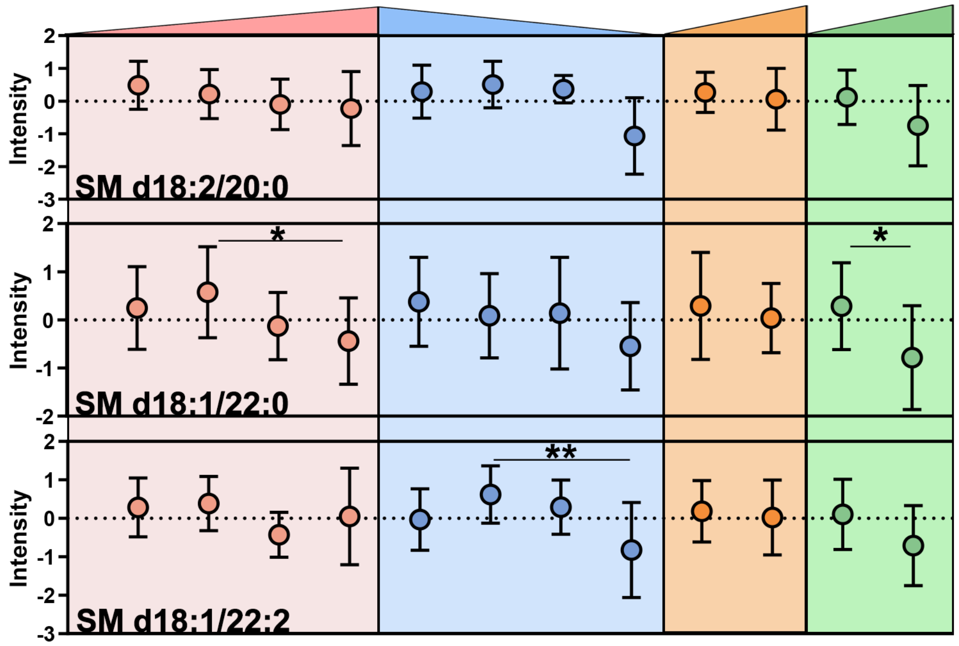

Figure S2. Common SM trend with either CRP (pink), P/F ratio (blue), D-dimer (orange) and creatinine (green) stratifications. Data are expressed as mean ± SD. Statistical significance is evaluated by one-way ANOVA with Bonferroni post-hoc test. P value are schematized as follows: * <0.05; **<0.01; *** < 0.001; ****<0.0001.
